# Fact or hypothesis: *Taenia crassiceps* as a model for *Taenia solium*, and the S3Pvac vaccine

**DOI:** 10.1111/j.1365-3024.2010.01231.x

**Published:** 2010-11

**Authors:** M W Lightowlers

**Affiliations:** Faculty of Veterinary Science, The University of MelbourneWerribee, Vic., Australia

**Keywords:** cestode, immunity, S3Pvac, Taenia crassiceps, vaccination

## Abstract

*Research undertaken over the past 40 years has established many of the general principals concerning immunity to taeniid cestodes. Although much is well understood about the host-protective mechanisms against taeniids and this knowledge has been exploited in studies on vaccine development, many aspects require further investigation or confirmation. Some phenomena have come to be regarded as being well established, while careful analysis of the published data would suggest that they may be better regarded as hypotheses rather than established facts. This review considers one selected issue pertaining to immunity to cestode infections and examines carefully the nature of the evidence that is available to support conclusions that have been made in this area. The issue examined is the use of* Taenia crassiceps *as a model for cysticercosis in pigs caused by* Taenia solium*, together with the S3Pvac vaccine, which has been developed based on this model. Strong evidence is found to support the conclusion that defined* T. crassiceps *antigens can limit intraperitoneal proliferation of the ORF strain of* T. crassiceps *in mice; however, the potential for these antigens to affect* T. solium *infection in pigs requires further confirmation*.

## Introduction

Taeniid cestodes are unusual parasites in that they seem to have an Achilles' heel in relation to their susceptibility to host immune attack. Intermediate hosts of many taeniid cestode parasite species have been shown to develop immune responses following an initial infection that act to prevent the establishment of parasites from a subsequent challenge infection while not affecting the parasites establishing from the initial exposure. This type of immunity, sometimes referred to as concomitant immunity, acts on the infective parasite stage, the oncosphere or early post-oncospheral stages (1). The existence of this Achilles’ heel has been exploited in the development of a series of highly effective recombinant vaccines based on oncosphere antigens (2). However, oncospheres are not the only life cycle stage that can be shown to be a source of host-protective antigens in taeniid cestodes (3). Also, there is clear evidence for antigenic cross-reactivity, with antigens from one species being capable of inducing host-protective responses against a related taeniid species [see, for example (4)]. These characteristics of the taeniid cestode parasites have encouraged some researchers to investigate host-protective antigens from sources other than oncospheres and to source protective antigens for practical use from model parasite species. One such research effort has involved the use of *Taenia crassiceps* in mice as a model for development of a vaccine against *Taenia solium*.

A recent review identified a number of topics concerning immunity to taeniid cestode parasites where, despite substantial data have been published in the area, either the issues remained unclear or commonly held interpretations were not clearly supported by published evidence (1). The usefulness of *T. crassiceps* as a model for *T. solium* and the S3Pvac vaccine developed using this model was one area identified as worthy of careful analysis. This forms the topic of the following review.

*Taenia crassiceps* has been used in an extensive series of experiments as a source of antigens and as a model for immunological studies concerning immunity to cysticercosis. Much of this work has been undertaken at the Instituto de Investigaciones Biomédicas, Universidad Nacional Autónoma de México, where *T. crassiceps* has been used as a model for *T. solium* infection in pigs. The work has led to the development of a synthetic peptide vaccine for *T. solium* based on epitopes selected from *T. crassiceps* proteins (5,6). From information contained in the various publications, this vaccine, termed S3Pvac, protects mice against infection with *T. crassiceps* and can be delivered as an effective vaccine as a synthetic peptide or via a novel bacterial vector or in transgenic plant tissue extracts or via expression on recombinant bacteriophage. When delivered in extracts from a recombinant bacterial vector, the vaccine is effective when given orally as well as parenterally. The vaccine also cross-protects pigs against infection with *T. solium* and has a therapeutic effect on parasites in infected pigs causing them to succumb to host inflammatory responses. In addition, the vaccine protects against infection with the adult tapeworm stage of *T. solium* in a laboratory model of infection. These attributes seem likely to qualify S3Pvac as the most robust of all vaccines in the field of parasitology, and possibly more broadly. Development of the S3Pvac vaccine has been reviewed elsewhere (5–7). The purpose of the following discussion is to examine the strength of the data provided in support of the reported effects of the S3Pvac vaccine and to consider *T. crassiceps* as a model parasite for studies on immunity to cysticercosis.

Prior to commenting on the use of *T. crassiceps* as a model for *T. solium* and the S3Pvac vaccine, it is important to point out what could be regarded as a conflict of interest. The author’s laboratory is engaged in research that seeks to achieve a similar outcome to the research being discussed, i.e. the development of a vaccine against *T. solium* infection in pigs. The discussion below concentrates on aspects relating directly to vaccination, that is protection *in vivo* against a parasite challenge. It does not consider data derived from various *in vitro* immunological investigations or antigen characterizations.

## *Taenia crassiceps* as a model for *Taenia solium*

*Taenia crassiceps* is a natural parasite of rodents and is transmitted by canines as definitive hosts. In the intermediate host, the parasite develops as a cysticercus in the abdominal cavity, where it proliferates. An animal infected with a single egg can potentially develop a heavy burden of cysticerci through proliferation of the parasite in the peritoneum. Few taeniid parasites have a similar biology in their intermediate hosts. The parasite’s site of election in the intermediate host is similar to *T. pisiformis* in rabbits and *T. hydatigena* in sheep but not to *T. solium* that predominantly encysts in the striated muscles of pigs and does not proliferate. The biology of *T. solium* seems to be more similar to *T. ovis* in sheep and *T. saginata* in cattle, or even *T. taeniaeformis* in rodents, than it is to *T. crassiceps*. Phylogenetic studies based on a variety of comparative assessment criteria place *T. ovis* and *T. saginata* close to *T. solium* and have *T. crassiceps* as a more distant relative (8–10).

The particular strain of *T. crassiceps* that has been used as a model for *T. solium* is the ORF strain. *T. crassiceps* was originally isolated from a fox and has been maintained for some 60 years by direct intraperitoneal passage of parasites between mice (11). After a number of years of passage, it was noticed that a particular line of parasites was growing more quickly in the mice and this rapidly growing parasite was designated the ORF strain (12). Biological investigations into this strain have uncovered some interesting characteristics in comparison the wild-type parasite. It can no longer infect the definitive host, has biochemical and immunological differences with the wild-type parasite (11,13) and is aneuploid (14), having lost one pair of chromosomes during the years of direct mouse-to-mouse passage. It is possible that parasite defence mechanisms against host immunological attack were lost along with the two chromosomes. This may limit the applicability of the ORF strain of *T. crassiceps* as a model for immunological studies of immunity to cysticercosis. *T. crassiceps* is an attractive laboratory model because it infects mice and can be maintained without recourse to passage through the definitive host.

## Vaccination with *Taenia crassiceps* parasite extracts

Antigenic cross-reactivity among taeniid cestodes is a widely recognized phenomenon. Antigenic preparations made from many species can be used as a source of host-protective antigens against a challenge infection with a heterologous species (15) or as a source of antigens for diagnosis of infection with a heterologous cestode (16). Consistent with this general observation, antigens obtained directly from *T. crassiceps* can be used as a source of material for detection of antibodies raised by infection with *T. solium* (17), and partial protection can be achieved against *T. solium* infection in pigs using antigens obtained from *T. crassiceps* (18) or *vice versa* (19).

Investigations utilizing *T. crassiceps* as a model and source of host-protective antigens for use against *T. solium* infection in pigs have followed a general path where vaccination experiments were performed initially in mice against an intraperitoneal infection with *T. crassiceps* and, having obtained encouraging data in the mouse model, similar experiments were undertaken in pigs against an experimental challenge infection with *T. solium*.

In 1990, Sciutto *et al*. (19) showed that extracts from *T. solium* cysticerci could limit the proliferation of the ORF strain of *T. crassiceps* in mice and concluded that *T. crassiceps* was a convenient laboratory model in which to test promising *T. solium* antigens for vaccine development. Valdez *et al*. (20) sought to fractionate *T. crassiceps* antigen extracts to identify individual protective antigens. They identified SDS–PAGE fractions comprising antigens of 56, 66 and 74 kDa, which, when used together as a vaccine, limited the proliferation of the ORF strain of *T. crassiceps* in vaccinated mice, achieving a 74% reduction in cysticerci numbers in comparison to nonimmunized controls. Interestingly, the authors noted one antigen fraction that induced statistically greater level of infection compared to controls.

Subsequently, Sciutto *et al*. (18) vaccinated pigs with a total extract of *T. crassiceps* cysticerci and challenged them with *T. solium*. Statistically significantly fewer cysticerci (58% reduction) of *T. solium* were found developing in muscle tissues compared to adjuvant-only (Freund’s Complete Adjuvant) controls; also, controls had more viable parasites (99%) than vaccinates (88%) (18). Vaccination with the same antigen preparation using alum as adjuvant led to a statistically significant *increase* in the number of cysticerci in the pigs. Pigs vaccinated with *T. crassiceps* extract using alum adjuvant had 37% cysticerci that were damaged, while the control group had 15·9% damaged parasites, suggesting that the effect on cyst viability that was noted when Freund’s Complete Adjuvant was used was either adjuvant-specific or not a reliable effect. With data from a single experiment and where another vaccinated group showed no protection, it is difficult to form a reliable conclusion about the ability of *T. crassiceps* cysticercus extract to protect pigs against *T. solium* infection.

Huerta *et al*. (21) provide further information on the use of *T. crassiceps* antigens in pigs. A vaccine trial using *T. crassiceps* cysticercus extract was carried out in rustic pigs, being animals sourced from rural areas rather than from a large commercial breeder. Sciutto *et al*.’s (18) finding that vaccination with *T. crassiceps* extract led to a reduction in the number of *T. solium* cysticerci following a challenge infection was not replicated. However, Huerta *et al*. (21) did present evidence to suggest that there was an effect of vaccination on the degree of inflammation seen in vaccinated pigs compared to controls, supporting the conclusions made by Sciutto *et al*. (18) that the vaccine increased the amount of inflammation around cysts. Huerta *et al*. (21) vaccinated pigs at either 40 or 70 days of age and necropsied them 80 days after challenge infection (110 days after first vaccination). Data were collected histologically on 694 parasites concerning the degree to which there was inflammation associated with each parasite, according to a five-point scale. The paper indicates that there was statistically significantly more inflammation in the parasites examined from vaccinated animals compared with controls; however, it is not possible to confirm the conclusion because the paper does not indicate how many parasites were examined from each animal, only the total number examined. Whether those that were examined were a statistically representative sample cannot be judged. Taken together, further investigations would be required to confirm whether *T. crassiceps* extracts have an ability to reduce either the number or viability of *T. solium* cysticerci in vaccinated pigs.

Manoutcharian *et al*. (22) refer to the results of an experiment where pigs were vaccinated with SDS–PAGE gel cut-outs containing the 56, 66 and 74 kDa antigens of *T. crassiceps*, in a similar fashion to the trials that had previously been conducted in mice (20). Full details of the experiment were provided in a subsequent paper (23). Seven pigs were vaccinated with the gel cut-outs. Of the seven vaccinated animals, only one animal had a single parasite. By comparison, seven control animals were infected with 3–7 parasites. The parasite burdens in this experiment were low; however, the differences between the groups are statistically significant, and the experiment does suggest that the gel slices containing *T. crassiceps* antigens may have a protective effect in pigs against infection with *T. solium*.

In the experiment discussed earlier in which pigs were vaccinated with gel cut-outs containing various fractions from a parasite extract, the control animals were immunized with adjuvant alone rather than gel cut-outs containing no antigen. The authors indicate that this was performed ‘considering previous observations that acrylamide does not modify the number of parasites recovered after infection’. What previous observations the authors were referring to is unclear as there appears to be no other publication where pigs had been vaccinated with polyacrylamide and subsequently challenged with *T. solium*. Similar experiments in mice (20) used nonimmunized animals as controls.

## The importance of experimental controls

In the publications discussed earlier and in a number of others discussed later, control groups were either untreated or given saline or adjuvant alone. A number of experiments do not include a placebo incorporating similar test substances while excluding the specific antigenic preparation being investigated. This does not detract from conclusions about the effects of the vaccination *per se*; however, it does raise doubts about confidence that observed effects are directly related to the antigen being tested and are not nonspecific. A number of situations have been described where statistically significant, nonspecific protection has been observed against cysticercosis in mice, for example following the injection of control DNA samples (24) or bacterial extracts (25) or plant extracts (26); nonspecific effects have achieved statistically significant levels of protection as high as 87%. Similar nonspecific protection has been obtained with another cestode parasite (27). It may seem unlikely that preparations such as synthetic peptides or polyacrylamide gel extracts would provide nonspecific protection against either *T. crassiceps* in mice of *T. solium* in pigs; however, this possibility remains in the absence of data to the contrary. Controls of this type do *not* need to be included in situations where previous experiments have included the appropriate controls for nonspecific effects, establishing clearly the vaccine’s efficacy and specificity. However, it is important that, at least in initial experiments, appropriate control preparations are included so that the effects can be clearly demonstrated to relate to the antigen being tested and are not nonspecific. This is particularly important where the effects of vaccination are modest and potentially within the bounds of known nonspecific effects within this group of parasites.

## Vaccination with recombinant antigens

Manoutcharian *et al*. (22) provide the first mention of cDNA cloning of proteins from *T. crassiceps* and vaccination of mice with different recombinant antigens designated KET; a later paper provided full details (23). A *T. crassiceps* cDNA library was screened for clones expressing antigens recognized by antisera raised against the 56 and 74 kDa antigens of *T. crassiceps* cysticerci. Positive clones were subsequently rescreened for reactivity with sera from pigs infected with *T. solium*. Five clones were found to be positive with the pig sera, and these were designated KETc1, KETc4, KETc7, KETc11 and KETc12. The DNA sequence of KETc7 was determined. The other clones were found to have cDNA inserts ranging from 0·4 to 1·4 kb. Sequencing of the 3′ approximately 400 bp of each cDNA suggested that the inserts were unrelated, although antibody affinity purified with the clones indicated that some clones showed immunological cross-reactivity. Crude lysates of the clones were used to vaccinate, mice and KETc1, KETc4, KETc7 and KETc11 were all shown to significantly reduce the proliferation of cysticerci of the ORF strain of *T. crassiceps* compared to controls. KETc11 significantly increased the level of parasite proliferation in the vaccinated animals.

Rosas *et al*. (28) cloned the fragment encoding the KETc7 protein into a DNA vaccine vector and used this to vaccinate mice. The results of the vaccine trial work are difficult to decipher in some respects, with the paper referring to the use of 10 mice per vaccine group, or elsewhere 8 per group, while referring to the results of one group and indicating that there were only 5 animals in the group. The vaccine did not induce a statistically significant reduction in *T. crassiceps* compared to controls receiving pcDNA3 DNA. The authors refer to a significant reduction in the proliferation of the ORF strain of *T. crassiceps* in mice receiving the KETc7 DNA vaccine construct compared with the results of mice treated with saline alone. In a subsequent paper, Cruz-Revilla *et al*. (29) obtained similar results. Vaccination of mice with a DNA vaccine expressing the KETc7 protein did not induce a statistically significant reduction in parasites in comparison with pcDNA3-treated controls. The authors indicate that there was a statistically significant difference if they compared the KETc7 group with saline-treated controls.

## Vaccination with selected antigenic epitopes

A computer-based epitope prediction software was used to predict antigenic regions of the KETc7 protein, and three of these were prepared as synthetic proteins and designated GK-1, GK-2 and GK-3 (30). These peptides were shown to react with antibodies in the sera of mice infected with *T. crassiceps* and, although Gevorkian *et al*. (30) refer to the peptides having reactivity with some sera from human cases of neurocysticercosis, it is unclear whether there is a significant difference between the levels shown and those seen with control sera. The peptides did not react with sera from pigs infected with *T. solium*. A follow-up paper (31) provides the results of a vaccination experiment in mice against a challenge infection with *T. crassiceps* involving each of the three peptides, together with a second trial using GK-1 only. In both trials, mice vaccinated with 50 μg of GK-1 had fewer cysticerci than mice immunized with adjuvant only. The authors indicate that they believed GK-2 and GK-3 provided no protection. In this case, the GK-2 and GK-3 groups could possibly act as ‘irrelevant’ peptide controls for comparison with the results of GK-1 (rather than using the adjuvant-only controls). However, mice vaccinated with GK-2 or GK-3 also developed fewer cysticerci than controls and *t*-test comparisons of parasite numbers in the groups vaccinated with GK-2 or GK-3, and those vaccinated with GK-1 show no significant differences. A second vaccination trial using GK-1 as either free peptide or conjugated to carrier proteins provided further support to the potential protective efficacy of the GK-1 peptide; however, control mice were immunized with adjuvant alone (saponin on this occasion).

Toledo *et al*. (32) extended the work on computer-predicted antigenic epitopes of the KET proteins. One epitope was predicted from the protein sequence of KETc1 and one also from KETc12. With the exception of the paper by Huerta *et al*. (33), the research group’s subsequent publications use the nomenclature KETc1 and KETc12 to refer to these 12 and 8 amino acid peptides, respectively, rather than the recombinant antigens originally given the same designations by Manoutcharian *et al*. (23). Three vaccine trials were conducted in mice using each of the KETc1 or KETc12 peptides against a challenge infection with *T. crassiceps*. In each case, a statistically significant reduction was observed in mice vaccinated with KETc1 (67–89% reduction) and KETc12 (57–70% reduction). Control mice did not receive an irrelevant peptide. Protection with the KETc1 peptide was confirmed by Sciutto *et al*. (25) in another trial in mice. While it is clear that the KETc1, KETc12 and GK-1 peptide *preparations* induce a significant reduction in *T. crassiceps* infection in mice, the *specificity* of those reductions in relation to the KET and GK-1 peptides has not been demonstrated clearly.

A novel expression system was evaluated for the delivery of the KETc1 peptide. The group describes the creation of a *Brucella* spp vector in which peptide epitopes can be expressed as part of the bacterial lumazine synthase protein (25,34). This protein assembles into stable dimers of pentamers, potentially providing an excellent epitope delivery system. The 12 amino acid peptide KETc1 was cloned into the vector, and mice were immunized with either the nonrecombinant bacterial lumazine synthase, lumazine synthase incorporating the KETc1 peptide, peptide alone or nonimmunized. Immunization with KETc1 peptide induced a statistically significant reduction in the proliferation of the ORF strain of *T. crassiceps* in vaccinated mice in comparison to nonimmunized controls. Mice immunized with the lumazine synthase incorporating KETc1 also developed significantly fewer cysticerci compared with the unimmunized controls. Mice immunized with the nonrecombinant lumazine synthase revealed a significant nonspecific reduction in number of cysticerci (57% reduction). Comparison between mice immunized with lumazine synthase and those immunized with lumazine synthase incorporating the KETc1 peptide revealed a reduced number in those receiving the construct incorporating the KETc1 peptide (*P* = 0·034, *t*-test; *P* = 0·082; Mann–Whitney *U*-test; *P* = 0·131, Tukey *t*-test). Rosas *et al*. (34) undertook follow-up experiments, which confirmed both the nonspecific protection with wild-type lumazine synthase and also an additional level of protection with lumazine synthase incorporating KETc1. Data in these two papers support the conclusion that the KETc1 peptide expressed with lumazine synthase can induce protection against *T. crassiceps* infection in mice specifically associated with the KETc1 peptide component.

Huerta *et al*. (33) undertook a vaccination trial in pigs using a combination of KETc1, KETc12 and CK-1 peptides against infection with *T. solium*. The trial was undertaken in the field against a natural challenge infection. The peptide combination used here has been referred to in some subsequent publications as S3Pvac (synthetic three peptide vaccine). Huerta *et al*. (33) adopted a strategy designed to take account of the potentially heterogeneous distribution in risk of exposure of the different experimental animals (35–37). A total of 140 control pigs and 138 vaccinated pigs were distributed in pairs amongst 70 different households. Vaccinated pigs received two immunizations with the three peptides plus saponin adjuvant at 60 and 90 days of age. Control pigs received saponin alone. Pigs were subjected to examination at necropsy between 10 and 12 months of age. At the completion of the trial, 18 vaccinated and 20 control pigs had died from causes unrelated to the experiment. Significantly fewer animals were infected in the vaccinated group (9 of 120) compared with the control group (19 of 120). Comparison of the burdens of infection in the two groups revealed a significant difference. A larger proportion of cysts in vaccinated animals were found to be necrotic (41%) compared to the proportion in controls (3·9%); it is not clear whether this difference was statistically significant. Based on the total number of parasites in the vaccinated and control groups, the authors calculated that the vaccine provided a 97·9% reduction in parasites. This conclusion is not supported by a statistical comparison of cyst numbers in the *infected* animals in the two groups (*P* = 0·534, Mann–Whitney *U*-test). Four animals in the control group had >10 000 cysts, while the highest number of parasites in a vaccinated animal was 1286, and this difference accounted for most of the 97·9% difference in total cyst numbers between the groups. The experimental design involved data pairs (vaccinated and control animals distributed in pairs to different households). The results were not analysed or provided as paired data. During the trial, 38 pigs became unavailable for necropsy; it seems likely that there were households where one member of a pair was unavailable at the time the necropsies were performed. The paper does not provide information about infection levels in the animals in relation to whether one member or both members of a pair were examined. How many, if any, of the heavily infected control animals were from households where the associated vaccinated animal was unavailable for necropsy cannot be determined.

Two further field trials have been undertaken with S3Pvac or variations of it. One utilized the same peptides as those used by Huerta *et al*. (33) and was undertaken in the State of Morelos, México (38). A total of 381 piglets were included in the trial, 32 of which were vaccinated with adjuvant alone, 95 received one injection of the S3Pvac and 254 received two immunizations with S3Pvac. At the time the pigs were necropsied, 56% of the experimental animals could not be recovered, leaving 20 controls, 48 in the group receiving one immunization and 98 in the group receiving two immunizations. The proportion of infected animals (2/20, 2/48 and 3/98, respectively) suggested a possible positive effect of the vaccine, but statistical comparisons were not significant. The authors made statistical comparisons after incorporating additional data from what were termed ‘sentinel pigs’. These animals were examined for *T. solium* infection at times either before or after the field trial was undertaken. Information provided about these animals is limited; it is unclear whether including these animals as additional controls in the analyses could be supported. However, after incorporating these animals, comparisons with the vaccinated groups revealed statistically significant protection by the vaccine. In conclusion, the field trials described by Huerta *et al*. (33) and Sciutto *et al*. (38) provide some evidence to suggest a positive effect of the S3Pvac vaccine in reducing the level of cysticercosis in vaccinated pigs; however, they do not provide compelling evidence for this conclusion.

Noting that a greater proportion of *T. solium* cysticerci in S3Pvac-vaccinated pigs were necrotic compared with controls in the field trial undertaken by Huerta *et al*. (33), de Aluja *et al*. (39) undertook an experiment in pigs to test the hypothesis that the vaccine had a cysticidal effect. Groups of five pigs were experimentally infected with *T. solium* and, 1 month later, vaccinated with either adjuvant alone or the CK-1 peptide or S3Pvac. Cysts were counted and assessed for the level of cyst-associated inflammation 8 months after the experimental infection. The paper indicates that there was a statistically significant reduction in the number of cysticerci in the muscles in S3Pvac-treated pigs vs. the number in controls (total 1039 cysts in controls; 563 in vaccinates) and a significantly increased percentage of damaged (nonvesicular) cysticerci in the muscles of S3Pvac-treated pigs compared to controls (total 61% damaged cysts in controls; 6% in vaccinates). de Aluja *et al*. (39) do not provide information about their statistical methods; examination of the data in the paper by Mann–Whitney *U*-test finds no statistically significant difference between S3Pvac-immunized pigs and controls with respect to either the total number of cysticerci or the percentage of damaged cysticerci. de Aluja *et al*. (39) include data obtained in *in vitro* measures of cyst viability on what were described as a ‘representative sample’ of cysticerci from the muscles, and these data supported the authors’ conclusion that the S3Pvac had a therapeutic effect on *T. solium* cysticercosis in vaccinated pigs. No information is provided about what was meant by the term ‘representative sample’, and data on cysts from individual animals were not provided. In summary, it is difficult to concur with the authors’ conclusion that S3Pvac has a (statistically) significant therapeutic effect on *T. solium* infection in pigs, based on the information provided in the paper.

In an adaptation of the S3Pvac, nucleotide sequences encoding the peptides comprising S3Pvac (KETc1, KETc12 and GK-1), as well as the entire KETc7 protein, were cloned by Manoutcharian *et al*. (40) into either the PIII protein or coat protein VIII of the filamentous bacteriophage M13. A mixture of phage expressing this antigen combination was termed CPLV (anti-cysticercotic phage vaccine). Data are provided about the effect of using this recombinant phage to vaccinate mice against *T. crassiceps* and pigs against *T. solium*. The results of the vaccine trial in mice are only given as the total number of cysts in controls given nonrecombinant phage (990 cysts) and the total number in vaccinates (338 cysts). This difference was indicated as ‘significant’ although what this indicates is unclear. The phage vaccine was tested in three pigs given the phage subcutaneously, three given the phage orally and five controls injected subcutaneously with nonrecombinant phage. All three pigs vaccinated with phage expressing the KET antigens had low numbers of *T. solium* cysticerci after the challenge infection (range 0–36) compared to the controls (range 91–580). Those receiving the phage orally showed a mixed response in terms of the number of parasites (31, 53 and 474 cysticerci). The authors noted that in three of the five infected animals that received the recombinant phage either subcutaneously or orally and were infected with cysticerci (one animal had no cysts), the majority of the cysticerci were necrotic. When data from the pigs receiving recombinant phage orally and subcutaneously were combined and compared with the controls, the percentage of necrotic cysticerci in the vaccinated group(s) was statistically significantly greater than that in the controls. The trial did not include a sufficient number of pigs in the vaccine groups to allow a confident conclusion to be drawn about the possible effects on either cyst numbers or cyst viability, although taken as preliminary data they could be considered encouraging in these respects.

Hernández *et al*. (26) describe an alternative methodology for the production of the KETc1, KETc12 and GK-1 peptides to the methods used in the previous studies. Oligonucleotides encoding these peptides were cloned into an embryonic cell line of the papaya plant. Extracts were prepared from recombinant plant callus tissue derived from a number of different clones expressing each of the peptides individually and the material used to vaccinate mice against a challenge infection with *T. crassiceps*. In two of the three experiments described, control mice immunized with extracts from nonrecombinant plants showed a statistically significant reduction in the number of cysticerci following the challenge infection compared with unimmunized controls (68% reduction and 80% reduction in the two experiments, respectively). Comparisons between the results obtained with one KETc7 clone and with several clones expressing KETc1 or KETc12 showed statistically significantly reduced numbers of *T. crassiceps* following the challenge infection compared with mice immunized with the extracts from the nonrecombinant plants. Several of the groups vaccinated with the extracts incorporating the KET peptides had the majority of the vaccinated animals completely free of parasites. Two groups immunized with extracts from clones expressing the KETc12 peptide had 98% fewer parasites than the mice injected with the nonrecombinant plant extract. It would appear that the KET peptides delivered in this way are superior to the synthetic peptides in inducing protection against *T. crassiceps* in mice. It is noteworthy that up to 80% nonspecific protection is seen in mice vaccinated with nonrecombinant plant extract. Nevertheless, the levels of cyst reduction seen in mice vaccinated with the KET peptides expressed in the plant extracts were repeatedly greater than the level of nonspecific reduction seen with control plant extracts, and hence, there is strong evidence to support the efficacy of the KETc1 and KETc12 peptides, in particular, in reducing the level of *T. crassiceps* proliferation in mice.

The most recent field trial undertaken to assess the potential for *T. crassiceps* antigens or epitopes to protect pigs against *T. solium* infection was that of Morales *et al*. (41) that describes the third in the series of field trials of the S3Pvac vaccine. The trial was carried out in the State of Puebla in México. The vaccine used was the same mix of four recombinant bacteriophages that were developed by Manoutcharian *et al*. (40).

There is some confusion in the nomenclature being used for the various vaccines in different papers. Prior to the publication by Morales *et al*. (41), all references to ‘S3Pvac’ had been to a combination of three synthetic peptides (KETc1, KETc12 and CK-1), also being the peptide combination used in the group’s two previous field trials. Manoutcharian *et al*. (40) refer to the combination of the four bacteriophage incorporating KETc1, KETc12 and CK-1 peptides together with the full KETc7 protein, as CPLV (anti-cysticercotic phage vaccine). However, Morales *et al*. (41) use the S3Pvac term to refer to a combination of bacteriophage, the same combination that Manoutcharian *et al*. (40) had earlier referred to as CPLV.

In their field trial, Morales *et al*. (41) vaccinated half of each litter of piglets used in the study and half acted as controls. The vaccine comprised a combination of 1 × 10^12^ of each of the four bacteriophage types; control pigs were vaccinated with saline. There were 626 vaccinated animals and 421 control animals at the beginning of the trial; necropsy data were collected from 134 controls and 197 vaccinated animals examined between 5 and 27 months after the start of the trial. Vaccination was shown to statistically significantly reduce the proportion of infected pigs (6·6% in vaccinates and 14·2% in controls) as well as the number of cysticerci counted at necropsy (mean number of cysts per community 8·4 cysts in infected vaccinates and 65·5 cysts in controls). These data provide evidence that the vaccine used in this study can reduce both the prevalence (54%) and intensity (87%) of *T. solium* infection in a field situation. While the data are strong to indicate that the bacteriophage vaccine provided significant protection against *T. solium* infection in this field trial, it is not clear that the effect was associated with the KET peptide component. Control animals received an immunization with saline alone. It has not been established that there is no nonspecific effect on the susceptibility of pigs to *T. solium* infection in pigs immunized with 4 × 10^12^ bacteriophages. The control animals in the experiment undertaken by Manoutcharian *et al*. (40) did receive an injection with nonrecombinant bacteriophage, and all animals became infected with *T. solium* cysticerci following the challenge infection, but it is not known whether the number of parasites establishing in these animals would have been the same in animals not immunized with the control bacteriophage. As mentioned previously, substantial nonspecific effects of up to 87% protection have been described for various control vaccine preparations when used in mice against *T. crassiceps*.

Wu *et al*. (42) have described the creation of a hepatitis B core protein fusion incorporating the KETc1, KETc12 and GK-1 peptides. The sequence encoding the modified hepatitis B core protein sequence was cloned into the pVAX3.0 vector to create a DNA vaccine. This vaccine was used in pigs against an experimental challenge infection with *T. solium*. Control pigs received pVAC3.0 DNA. Pigs vaccinated with the DNA vaccine expressing the KET peptides as part of the hepatitis B core protein had statistically significantly fewer cysticerci development following the *T. solium* challenge infection compared with control animals (83% reduction). It can be concluded that the vaccine induced protection against *T. solium* infection, but the specificity of this protection in relation to the KET peptides would require confirmation. This is because of the KET test group received vector expressing hepatitis B core protein plus KET peptides, while the controls received a vector not expressing the hepatitis B core protein, and hence, the experiment was not controlled for potential nonspecific effects of hepatitis B core protein.

## Conclusions

In summary, the published evidence provides solid support for the following conclusions about the use of *T. crassiceps* antigens as vaccines against *T. crassiceps* in mice or *T. solium* in pigs:

- Native antigens of *T. crassiceps* and *T. solium* provide protection for mice against the proliferation of the ORF strain of *T. crassiceps*.
- KETc1, KETc4 and KETc7 recombinant proteins protect mice against *T. crassiceps*.
- KETc1 peptide protects mice against *T. crassiceps* when delivered as a *Brucella* lumazine synthase fusion protein.
- KETc1 or KETc12 peptides protect mice against *T. crassiceps* when prepared from transgenic papaya tissue.
- A mixture of 4 × 10^12^ bacteriophages comprising equal numbers expressing KETc1, KETc12 and CK-1 peptides and the KETc7 protein provides protection in pigs against a natural field infection with *T. solium*, although the specificity of this protection relating to the KET component of the vaccine, rather than the bacteriophage component, has yet to be established.

Taking into account the analyses outlined earlier, the available evidence, while not arguing against the following hypotheses, does not provide solid support for the following:

- Protection in pigs against an experimental infection with *T. solium* induced using native antigens from *T. crassiceps*.
- Protective efficacy of KETc7 in mice against *T. crassiceps* infection when delivered as a DNA vaccine.
- Protective efficacy of KET synthetic peptides in protecting mice against infection with *T. crassiceps* or protecting pigs against an experimental infection with *T. solium*.
- A therapeutic effect of the synthetic peptide vaccine S3Pvac against *T. solium* infection in pigs.
- A protective effect of the S3Pvac synthetic peptide vaccine against *T. solium* infection in pigs.

Further research is required using the S3Pvac vaccine to establish clearly the potential value of the vaccine for use against *T. solium* infection in pigs and to confirm that the effects described for the vaccine delivered as recombinant bacteriophage are associated with the antigen-specific component of the vaccine rather than the bacteriophage vector.

There has been an increase in interest in the potential for vaccination to play a role in control of *T. solium* transmission, with a number of research groups having described effective vaccines for use in pigs (43). It will be important that these vaccines are validated independently and that those shown to be reliable and effective are assessed for other attributes such as cost and ease of use in practical situations.

## References

[1] Lightowlers MW (2010). Fact or hypothesis: concomitant immunity in taeniid cestode infections. Parasite Immunol.

[2] Lightowlers MW (2006). Cestode vaccines: origins, current status and future prospects. Parasitology.

[3] Rickard MD, Williams JF (1982). Hydatidosis/cysticercosis: immune mechanisms and immunization against infection. Adv Parasitol.

[4] Gemmell MA (1964). Species specificity of the immunogenic complexes of the tapeworm hexacanth embryo. Nature.

[5] Sciutto E, Rosas G, Hernandez M (2007). Improvement of the synthetic tri-peptide vaccine (S3Pvac) against porcine *Taenia solium* cysticercosis in search of a more effective, inexpensive and manageable vaccine. Vaccine.

[6] Sciutto E, Fragoso G, Aluja ASd, Hernandez M, Rosas G, Larralde C (2008). Vaccines against cysticercosis. Curr Top Med Chem.

[7] Sciutto E, Fragoso G, Manoutcharian K (2002). New approaches to improve a peptide vaccine against porcine *Taenia solium* cysticercosis. Arch Med Res.

[8] de Queiroz A, Alkire NL (1998). The phylogenetic placement of *Taenia* cestodes that parasitize humans. J Parasitol.

[9] Hoberg EP, Jones A, Rausch RL, Eom KS, Gardner SL (2000). A phylogenetic hypothesis for species of the genus *Taenia* (Eucestoda : Taeniidae). J Parasitol.

[10] Hoberg EP (2006). Phylogeny of *Taenia*: Species definitions and origins of human parasites. Parasitol Int.

[11] Freeman RS (1962). Studies on the biology of *Taenia crassiceps* (Zeder 1810) Rudolphi, 1810 (cestoda). Can J Zool.

[12] Dorais FJ, Esch GW (1969). Growth rate of two *Taenia crassiceps* strains. Exp Parasitol.

[13] Fox LL, Kuhn RE, Esch GW (1971). *Taenia crassiceps*: antigenic comparison of two larval strains. Exp Parasitol.

[14] Smith JK, Esch GW, Kuhn RE (1972). Growth and development of larval *Taenia crassiceps* (cestoda). I. Aneuploidy in the anomalous ORF strain. Int J Parasitol.

[15] Lightowlers MW, Mitchell GF, Rickard MD, Warren KS, Agabian N (1992). Cestodes. Immunology and Molecular Biology of Parasitic Infections.

[16] Yong WK, Heath DD, van Knapen F (1984). Comparison of cestode antigens in an enzyme-linked immunosorbent assay for the diagnosis of *Echinococcus granulosus*, *Taenia hydatigena* and *T. ovis* infections in sheep. Res Vet Sci.

[17] Larralde C, Sotelo J, Montoya RM (1990). Immunodiagnosis of human cysticercosis in cerebrospinal fluid. Antigens from murine Taenia crassiceps cysticerci effectively substitute those from porcine Taenia solium. Arch Pathol Lab Med.

[18] Sciutto E, Aluja A, Fragoso G (1995). Immunization of pigs against *Taenia solium* cysticercosis: factors related to effective protection. Vet Parasitol.

[19] Sciutto E, Fragoso G, Trueba L (1990). Cysticercosis vaccine: cross protecting immunity with *T. solium* antigens against experimental murine *T. crassiceps* cysticercosis. Parasite Immunol.

[20] Valdez F, Hernandez M, Govezensky T, Fragoso G, Sciutto E (1994). Immunization against *Taenia crassiceps* cysticercosis: identification of the most promising antigens in the induction of protective immunity. J Parasitol.

[21] Huerta M, Sciutto E, Garcia G (2000). Vaccination against *Taenia solium* cysticercosis in underfed rustic pigs of Mexico: roles of age, genetic background and antibody response. Vet Parasitol.

[22] Manoutcharian K, Larralde C, Aluja A, Chanock AM, Brown F, Ginsberg HS, Norrby E (1995). Advances in the development of a recombinant vaccine against *Taenia solium* pig cysticercosis. Vaccine 95 Molecular Approaches to the Control of Infectious Diseases.

[23] Manoutcharian K, Rosas G, Hernandez M (1996). Cysticercosis: identification and cloning of protective recombinant antigens. J Parasitol.

[24] Cruz-Revilla C, Sonabend AM, Rosas G (2006). Intrahepatic DNA vaccination: unexpected increased resistance against murine cysticercosis induced by nonspecific enhanced immunity. J Parasitol.

[25] Sciutto E, Toledo A, Cruz C (2005). *Brucella* spp. lumazine synthase: a novel antigen delivery system. Vaccine.

[26] Hernandez M, Cabrera-Ponce JL, Fragoso G (2007). A new highly effective anticysticercosis vaccine expressed in transgenic papaya. Vaccine.

[27] Thompson RC, Penhale WJ, White TR, Pass DA (1982). BCG-induced inhibition and destruction of *Taenia taeniaeformis* in mice. Parasite Immunol.

[28] Rosas G, Cruz-Revilla C, Fragoso G (1998). *Taenia crassiceps* cysticercosis: humoral immune response and protection elicited by DNA immunization. J Parasitol.

[29] Cruz-Revilla C, Rosas G, Fragoso G (2000). *Taenia crassiceps* cysticercosis: protective effect and immune response elicited by DNA immunization. J Parasitol.

[30] Gevorkian G, Manoutcharian K, Larralde C (1996). Immunodominant synthetic peptides of *Taenia crassiceps* in murine and human cysticercosis. Immunol Lett.

[31] Toledo A, Larralde C, Fragoso G (1999). Towards a *Taenia solium* cysticercosis vaccine: an epitope shared by *Taenia crassiceps* and *Taenia solium* protects mice against experimental cysticercosis. Infect Immun.

[32] Toledo A, Fragoso G, Rosas G (2001). Two epitopes shared by *Taenia crassiceps* and *Taenia solium* confer protection against murine *T. crassiceps* cysticercosis along with a prominent T1 response. Infect Immun.

[33] Huerta M, de Aluja AS, Fragoso G (2001). Synthetic peptide vaccine against *Taenia solium* pig cysticercosis: successful vaccination in a controlled field trial in rural Mexico. Vaccine.

[34] Rosas G, Fragoso G, Ainciart N (2006). *Brucella* spp. lumazine synthase: a novel adjuvant and antigen delivery system to effectively induce oral immunity. Microbes Infect.

[35] Keilbach NM, de Aluja AS, Sarti-Gutierrez E (1989). A programme to control taeniasis-cysticercosis (*T. solium*): experiences in a Mexican village. Acta Leiden.

[36] Diaz Camacho SP, Candil Ruiz A, Suate Peraza V (1991). Epidemiologic study and control of *Taenia solium* infections with praziquantel in a rural village of Mexico. Am J Trop Med Hyg.

[37] Sarti E, Schantz PM, Plancarte A (1994). Epidemiological investigation of *Taenia solium* taeniasis and cysticercosis in a rural village of Michoacan state, Mexico. Trans R Soc Trop Med Hyg.

[38] Sciutto E, Morales J, Martinez JJ (2007). Further evaluation of the synthetic peptide vaccine S3Pvac against *Taenia solium* cysticercosis in pigs in an endemic town of Mexico. Parasitology.

[39] de Aluja AS, Villalobos NM, Nava G (2005). Therapeutic capacity of the synthetic peptide-based vaccine against *Taenia solium* cysticercosis in pigs. Vaccine.

[40] Manoutcharian K, Diaz-Orea A, Gevorkian G (2004). Recombinant bacteriophage-based multiepitope vaccine against *Taenia solium* pig cysticercosis. Vet Immunol Immunopathol.

[41] Morales J, Martinez JJ, Manoutcharian K (2008). Inexpensive anti-cysticercosis vaccine: S3Pvac expressed in heat inactivated M13 filamentous phage proves effective against naturally acquired *Taenia solium* porcine cysticercosis. Vaccine.

[42] Wu L, Diao Z, Deang X (2005). DNA vaccine against *Taenia solium* cysticercosis expressed as a modified hepatitis B virus core particle containing three epitopes shared by *Taenia crassiceps* and *Taenia solium*. J Nanosci Nanotechnol.

[43] Lightowlers MW (2010). Eradication of *Taenia solium* cysticercosis: a role for vaccination of pigs. Int J Parasitol.

